# Real‐world analysis of the prognostic value of *EGFR* mutation detection in plasma ctDNA from patients with advanced non‐small cell lung cancer

**DOI:** 10.1002/cam4.5582

**Published:** 2023-01-09

**Authors:** Chaolian Long, Kun Li, Zichen Liu, Nana Zhang, Xuya Xing, Liming Xu, Fei Gai, Nanying Che

**Affiliations:** ^1^ Department of Pathology, Beijing Chest Hospital Capital Medical University, Beijing Tuberculosis and Thoracic Tumor Research Institute Beijing China; ^2^ Amoy Diagnostics Co., Ltd Xiamen China

**Keywords:** *EGFR* mutation, NSCLC, plasma samples, prognosis, superARMS‐PCR

## Abstract

**Background:**

The plasma sample has emerged as a promising surrogate sample for *EGFR* mutation detection in advanced non‐small cell lung cancer (NSCLC). In clinical practice, whether *EGFR* variants in baseline plasma ctDNA of advanced NSCLC can predict prognosis in addition to guiding targeted therapy remains to be further explored.

**Material and Methods:**

In total, 315 NSCLC patients were retrospectively enrolled. *EGFR* mutation data from tissue detected by ARMS‐PCR and paired plasma samples within 1 month of admission detected by SuperARMS or ARMS‐PCR were collected. The correlation between baseline plasma ctDNA *EGFR* mutation status and survival was compared.

**Results:**

*EGFR* mutation detection rates in tumor samples and plasma samples were 65.1% (205/315) and 43.8% (138/315). Referred to tissue results, the consistent rate of test ctDNA *EGFR* alteration by SuperARMS was higher than that detected by ARMS (79.5% vs. 69.0%, *p* = 0.04), either in stage I–IIIA patients (85.7% vs. 50.0%, *p* = 0.4) or stage IIIB–IV patients (79.1% vs. 69.4%, *p* = 0.04). Patients' treatment status and pathological subtype were the two factors that affected plasma ctDNA *EGFR* alteration detection accuracy. The concordance in non‐adenocarcinoma patients was obviously higher than that in adenocarcinoma (*p* = 0.02), and the concordance in treatment naïve patients was significantly higher than that in relapse patients (*p* = 0.047). In treatment naïve patients, the median PFS (mPFS) in plasma ctDNA *EGFR*‐positive patients was shorter than that in plasma ctDNA *EGFR* negative patients (7.0 vs. 10.0 months, *p* = 0.01). In relapsed patients, the mPFS in plasma ctDNA *EGFR*‐positive patients was 9.0 months versus 11.0 months in plasma ctDNA *EGFR* negative patients (*p* = 0.1).

**Conclusions:**

A plasma sample could be an alternative for a molecular test when tissue samples was unavailable. The SuperARMS‐PCR detection method has high sensitivity in real‐world clinical practice. Furthermore, in patients with stage IIIB‐IV, baseline plasma ctDNA *EGFR* mutation positivity not only guides targeted therapy but also predicts a worse prognosis.

## Graphical abstract

Confiremed the concordance of test ctDNA *EGFR* alteration by Super‐PCR was higher than detected by ARMS‐PCR. And in ctDNA *EGFR* mutation positivity not only guide targeted therapy, but also predict a worse prognosis.

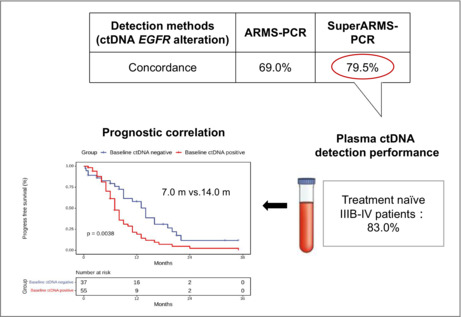

## 1 INTRODUCTION

Lung cancer has been the worst global burden of cancer according to the statistics from World Health Organization's International Agency for Research on Cancer, with a high incidence and high mortality, among which non‐small cell lung cancer (NSCLC) accounts for about 85% of all cases.^1^, ^2^ In recent years, with the development and clinical application of small molecule targeted drugs, the five‐year survival rate has been dramatically improved in NSCLC panties.^3^, ^4^ Epidermal growth factor receptor (EGFR) is one of the key target, which mutations drive about 50% of NSCLC in Asia.^5^ Advanced NSCLC patients with *EGFR* mutations occurring in exons 18–21 can dramatically benefit from EGFR tyrosine kinase inhibitors (TKIs) out of other drug therapies.^6^, ^7^ Since it is often difficult to obtain adequate tissue samples for *EGFR* testing in patients with advanced lung cancer, liquid biopsy has been the promising surrogate sample.^8^, ^9^ Liquid biopsy is an analytical diagnosis of diseases such as cancer by examining blood or other body fluids such as urine, saliva, pleural fluid, and cerebrospinal fluid.^10^ Plasma circulating tumor DNA (ctDNA) refers to tumor‐derived DNA fragments consisting of plasma‐free DNA (cfDNA).^11^ Multiple clinical studies have confirmed that the accuracy rate of plasma ctDNA *EGFR* mutation detection at baseline before treatment can reach 70%, and the guiding role of targeted drugs was consistent with tissue results.^12^, ^13^, ^14^

Current studies have also shown that ctDNA is a non‐invasive real‐time biomarker that can provide prognostic and predictive information for monitoring treatment.^15^, ^16^ The prognostic value of ctDNA has been demonstrated in recent years in detecting minimal residual disease after surgery or treatment.^17^, ^18^, ^19^ In addition, Plasma ctDNA has also been an indicator for tracking molecular residual disease (MRD) after radical treatment in patients with locally advanced lung cancer.^20^, ^21^, ^22^, ^23^ All of these studies confirmed the prognostic value of plasma ctDNA‐based assessment of MRD, i.e., no mutation was detected in plasms ctDNA within 1 month after curative treatment, and patients were more likely to have a longer DFS.^17^, ^20^, ^21^, ^24^ However, few studies have demonstrated that plasma ctDNA mutation status can predict the prognosis of advanced lung cancer. In a pan‐cancer analysis of immune checkpoint blockade, baseline ctDNA levels appeared prognostic value.^25^ In addition, a prospective NSCLC study showed that both ctDNA detected at baseline diagnosis and residual ctDNA identified at the first post‐treatment assessment were associated with poor prognosis.^15^ From the perspective of dynamic monitoring, ctDNA clearance is a marker of good prognosis. Patients with at least one ctDNA clearance have significantly longer OS and PFS than those with no ctDNA clearance.^26^

However, with different detection methods, ctDNA‐detected results in therapeutic monitoring may also be inconsistent. Currently, among the various blood *EGFR* mutation detection techniques, SuperARMS technology is a highly sensitive and specific method suitable for non‐invasive detection of advanced NSCLC patients with plasma *EGFR* mutation detection, the detection sensitivity of *EGFR* mutation detection in plasma samples can reach 0.2%, which is can effectively predict the efficacy of EGFR‐TKIs and guide clinical medication.^27^, ^28^, ^29^

In our study, we collected paired tissue and plasma samples to explore the consistency of plasma cfDNA *EGFR* variants. And further explored the prognostic value of ctDNA monitoring in advanced NSCLC therapies. In the present study, neither the molecular detection method including ARMS‐PCR or SuperARMS‐PCR nor the follow‐up treatment regimens have been intervened. The assay performance of the reagents thus evaluated may be more suggestive for clinical practice. The factors that could affect the detection rate of ctDNA EGFR mutation were deeply analyzed. The data from this study showed that the SuperARMS can be used to detect plasma samples from NSCLC patients in early stages, whereas previous data reported in the literature almost all focus on samples from patients in advanced stages.^28^, ^34^ Second, in the evaluation of the correlation between baseline ctDNA mutation status and patient prognosis, patients treated with targeted therapy, and chemotherapy were included in the analysis. This also distinguished itself from previous reports in which targeted therapies have been the mainstay of research.^15^, ^43^

## 2 MATERIALS AND METHODS

### 2.1 Patients and study design

**FIGURE 1:**
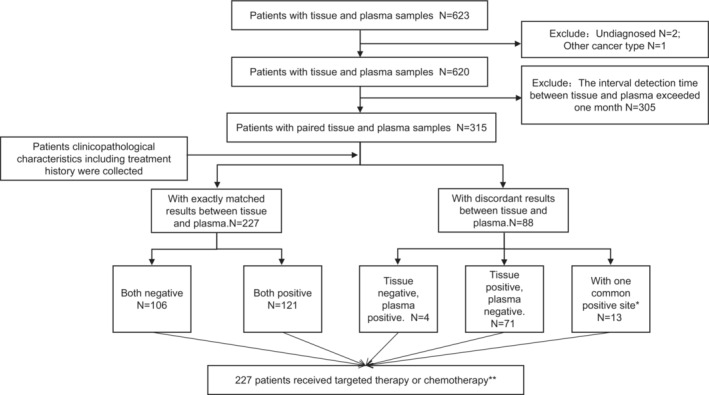
Flowchart of the study. *Tissue or plasma samples have been detected more than one EGFR mutation, and only one of them was detected in both kinds of samples. **150 patients received first‐ or second‐generation EGFR‐TKIs and 77 patients received chemotherapy.

From July 2016 to April 2021, 623 patients with both tissue and plasma *EGFR* mutation detection records were retrospectively screened in the Department of Pathology, Beijing Chest Hospital. Among them, the exclusion criteria were set as follows: (1) Patients who cannot be pathologically diagnosed with non‐small cell lung cancer, (2) The interval detection time between tissue and plasma exceeded 1 month. Therefore, 315 pathologically diagnosed NSCLC patients were finally enrolled in this study. The patients' clinicopathological information, including age, gender, tumor‐node‐metastasis (TNM) stage, smoking history, *EGFR* molecular detection results of tissue and plasma samples, and treatment history were investigated and collected in the clinicopathological system and pathological information system of our hospital (Figure 1). Of these 315 patients, ARMS‐PCR was used for plasma samples from June 2016 to April 2018 and SuperARMS‐PCR was used for plasma samples after April 2018 along with its approval by NMPA (National Medical Products Administration). Patients' survival follow‐up data were analyzed using July 2021, data cut‐off.

### 2.2 Tissue and plasma *EGFR* mutation detection

The *EGFR* mutation status of plasma samples was detected with ARMS or Super‐ARMS *EGFR* Mutation Detection Kit (Amoy Diagnostics) according to the manufacturer's instructions. *EGFR* mutation status of the tumor tissues was detected using the ARMS *EGFR* Mutations Detection Kit (Amoy Diagnostics) according to the manufacturer's instructions.

### 2.3 Statistics on progression‐free survival

Among the 315 enrolled patients, a total of 268 patients received chemotherapy or targeted therapy after plasma testing, and only 227 of 268 patients had relatively complete treatment follow‐up information, among which 139 treatment naïve patients and 82 relapsed patients were in stage IIIB‐IV. Patients' treatment efficacy data were collected and progression‐free survival (PFS) was calculated. PFS differences were compared between patients with positive and negative ctDNA *EGFR* mutation in plasma before treatment, regardless of subsequent treatment regimens.

### 2.4 Statistical analysis

The data were analyzed using SPSS software version 20 (IBM). The relationship between the two groups was evaluated using a standard chi‐squared test, and *p* < 0.05 was considered statistically significant. The Kaplan–Meier was used for PFS analysis, and survival curves were compared using the log‐rank test.

## 3 RESULTS

### 3.1 Patient characteristics and the *EGFR* mutation status in tissue and plasma samples

Among the 315 enrolled NSCLC patients, 149 (47.3%) were males, 166 (52.7%) were females, 193 (61.3%) patients were over 60 years old, 184 (58.4%) had never smoked; the number of treatment naïve patients were 232 (73.7%), 299 (94.9%) were in stage IIIB‐IV, and 279 (88.6%) had adenocarcinoma. The clinicopathological characteristics of the patients are shown in Table 1.

**TABLE 1:** Clinicopathological characteristics of enrolled 315 patients with non‐small cell lung cancer

Characteristics	All case (*N* = 315)	*n* (%)
Sex		
Male	149	47.3
Female	166	52.7
Age		
≤60	122	38.7
>60	193	61.3
Smoking status		
Yes	131	41.6
No	184	58.4
Treatment status		
Treatment naïve	232	73.7
Relapsed^a^	83	26.3
Stages		
I	7	2.2
II	5	1.6
IIIA	4	1.3
IIIB	15	4.8
IV	284	90.1
Pathological subtype		
Adenocarcinoma	279	88.6
Non‐adenocarcinoma	36	11.4

^a^ 82 of the 83 relapsed patients had comprehensive treatment follow‐up records. 57 patients relapsed from EGFR TKIs and 25 patients relapsed after adjuvant chemotherapy.

In the present study, *EGFR* mutation was detected in 205 (65.1%) tissue samples and 138 (43.8%) plasma samples. In tissue samples, a total of 237 *EGFR* mutation sites were detected, including L858R (95/237, 40.1%) and 19‐Del (93/237, 39.2%), T790M (29/237, 12.2%), G719X (9/237, 3.8%), 20‐Ins (7/237, 3.0%), and L861Q (4/237, 1.7%). In plasma samples, a total of 156 *EGFR* mutation sites were detected, including L858R (56/156, 35.6%), 19‐Del (65/156, 41.7%), T790M (18/156, 11.5%), G719X (8/156, 5.1%), 20‐Ins (5/156, 3.2%), and L861Q (4/156, 2.6%). The *EGFR* mutation profile in detail is shown in Figure S1.

### 3.2 Performance of plasma ctDNA *EGFR* detection

The total sensitivity, specificity, negative predictive value (NPV), and positive predictive value (PPV) of ctDNA detected by ARMS was 59.7% (95% CI: 38.9%, 66.1%), 92.9% (95% CI: 47.5%, 70.9%), 95.6% (95% CI: 83.6%, 99.2%), and 47.3% (95% CI: 33.9%, 61.1%). The total sensitivity, specificity, NPV, and PPV of ctDNA detected by SuperARMS were 68.4% (95% CI: 59.4%, 76.0%), 97.6% (95% CI: 90.6%, 99.6%), 97.8% (95% CI: 91.7%, 99.6%), and 65.6% (95% CI: 56.4%, 73.8%). Compared with *EGFR* mutation detection in tumor tissue DNA, the total concordance rate of ctDNA detected by SuperARMS was higher than that of ARMS (79.5% [95% CI: 73.7%, 84.4%] vs. 69% [95% CI: 62.0%, 80.3%], *p* = 0.04), especially in patients in stage I‐IIIA (85.7% [95% CI: 60.1%, 96.0%] vs. 50.0% [95% CI: 9.5%, 90.6%], *p* = 0.4) compared with patients in stage IIIB‐IV (79.1% [95% CI: 73.0%, 84.2%] vs. 69.4% [95% CI: 59.7%, 77.6%], *p* = 0.04). Besides, in treatment naïve patients the concordance of ctDNA detected by SuperARMS were higher than those ctDNA detected by ARMS (83.0% [95% CI: 76.3%, 88.1%] vs. 65.8% [95% CI: 54.9%,75.3%], *p* = 0.003). Reverse results were observed in relapsed patients with no significant difference (71.0% [95% CI: 58.7%, 80.8%] vs. 81% [95% CI:60.0%, 92.3%], *p* = 0.4) (Table 2).

**TABLE 2:** Concordance of *EGFR* alteration status between tissue and plasma

	Plasma ctDNA detected by ARMS^a^ (*N* = 100)					Plasma ctDNA detected by SuperARMS^b^ (*N* = 215)				
	Sensitivity (95% Cl)	Specificity (95% Cl)	PPV (95% Cl)	NPV (95% Cl)	Concordance (95% Cl)	Sensitivity (95% Cl)	Specificity (95% Cl)	PPV (95% Cl)	NPV (95% Cl)	Concordance (95% Cl)
Total	59.7% (38.9%, 66.1%)	92.9% (47.5%, 70.9%)	95.6% (83.6%, 99.2%)	47.3% (33.9%, 61.1%)	69.0% (62.0%, 80.3%)	68.4% (59.7%, 76.0%)	97.6% (90.6%, 99.6%)	97.8% (91.7%, 99.6%)	65.6% (56.4%, 73.8%)	79.5% (73.7%, 84.4%)
Stage										
I‐IIIA	0% (0, 94.5%)	100.0% (5.5%, 100%)	—	50.0% (0.3%, 97.3%)	50.0% (9.5%, 90.6%)	33.3% (1.8%, 87.5%)	100.0% (67.9%, 100.0%)	100.0% (5.5%, 100.0%)	84.6% (53.7%, 97.3%)	85.7% (60.1%, 96.0%)
IIIB‐IV	60.6% (48.2%, 71.7%)	92.6% (74.2%, 98.7%)	95.6% (83.6%, 99.2%)	47.2% (33.5%, 61.2%)	69.4% (59.7%, 77.6%)	69.2% (60.4%, 76.9%)	97.2% (89.3%, 99.5%)	97.8% (91.6%, 99.6%)	63.3% (53.4%, 72.2%)	79.1% (73.0%, 84.2%)
Treatment Naïve	52.8% (38.7%, 66.5%)	92.3% (73.4%, 98.7%)	93.3% (76.5%, 98.8%)	49% (34.6%, 63.5%)	65.8% (54.9%,75.3%)	67.1% (38.8%, 66.5%)	100.0% (73.4%, 98.7%)	100.0% (76.5%, 98.8%)	74.0% (34.6%, 63.5%)	83.0% (76.3%, 88.1%)
Relapsed	78.9% (53.9%, 93.0%)	100.0% (19.8%, 100.0%)	100% (74.7%, 100.0%)	33.3% (24.1%, 94.0%)	81.0% (60.0%, 92.3%)	70.4% (56.2%, 81.6%)	75.0% (35.6%, 95.5%)	95.0% (81.8%, 99.1%)	27.3% (11.6%, 50.4%)	71.0% (58.7%, 80.8%)

Abbreviations: NPV, negative predictive value; PPV, positive predictive value.

^a^ A total of 100 patients was detected by ARMS, two patients were in stage I‐IIIA and both were treatment naïve; 98 patients were in stage IIIB‐IV of whom 77 were treatment naïve and 21 were relapsed.

^b^ A total of 215 patients was detected by SuperARMS, 14 patients were in stage I‐IIIA and all were treatment naïve, and 201 patients were in stage IIIB‐IV of whom 139 were treatment naïve and 62 were relapsed.

The clinicopathological characteristics affecting the consistency between tissue DNA *EGFR* detection and plasma ctDNA *EGFR* detection by SuperARMS were analyzed. The results showed that there were statistically significant differences in treatment status and pathological types. Eight of the 28 non‐adenocarcinoma patients detected by SuperARMS had *EGFR* mutation, seven of which were positive both in tissue and plasma, one of which was only positive in tissue. The concordance in non‐adenocarcinoma patients was obviously higher than that in adenocarcinoma (*p* = 0.02) (Table S1). The concordance in treatment naïve patients was significantly higher than that in relapse patients (*p* = 0.047) (Table S2, Table 3). No statistical difference was observed in age, sex, smoking status, and tumor specimen stage (Table 3).

**TABLE 3:** Clinicopathological characteristics affecting the consistency between tissue and plasma

Characteristics	Consistency between tissue and plasma (*N* = 215^a^)		*χ* ^2^/*p* value
	Consistent	Inconsistent	
Sex			
Male	88	17	0.2
Female	85	25	
Age			
≤60	65	13	0.3
>60	106	31	
Smoking states			
Yes	72	19	0.9
No	99	25	
Treatment status			
Treatment naïve	127	26	<0.05*
Relapse	44	18	
Stage			
I‐IIIA	12	2	0.7
IIIB‐IV	159	42	
Pathological type			
Adenocarcinoma	144	43	0.02
Non‐adenocarcinoma	27	1	

^a^ The plasma ctDNA *EGFR* mutation was detected by SuperARMS.

* *p* = 0.047; *p* < 0.05: significant different.

### 3.3 Correlation between baseline plasma ctDNA *EGFR* mutation status and prognosis in advanced NSCLC patients

**FIGURE 2:**
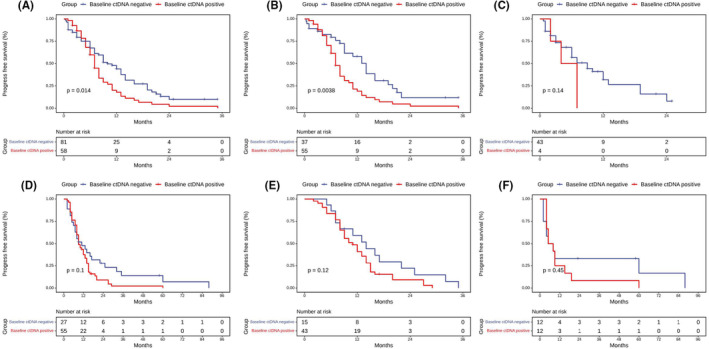
The progress‐free survival (PFS) of baseline ctDNA‐negative and ctDNA‐positive patients with advanced NSCLC. Comparison of PFS in treatment‐naive patients (A) and relapsed patients (D) regardless of the therapy regimen. Comparison of PFS with targeted therapy in treatment naïve patients (B) and relapsed patients (E). Comparison of PFS with chemotherapy in treatment naïve patients (C) and relapsed patients (F).

In 139‐treatment naïve IIIB‐IV patients, patients with baseline ctDNA *EGFR*‐negative had a significantly longer PFS than those of *EGFR*‐positive (10.0 months vs. 7.0 months, *p* = 0.01) regardless of treatment regimen (Figure 2A). Ninety‐two of 139 patients with tissue *EGFR*‐positive received first‐ or second‐generation EGFR‐TKIs treatment. The median PFS (mPFS) of the 37 patients with plasma ctDNA *EGFR*‐negative was significantly longer than that of the 55 patients with plasma ctDNA *EGFR*‐positive (14.0 months vs.7.0 months, *p* = 0.004) (Figure 2B). Forty‐seven of 139 patients with tissue *EGFR*‐positive received chemotherapy. The mPFS between ctDNA *EGFR*‐negative and *EGFR*‐positive patients in the targeted therapy was 7.0 months vs. 4.0 months (*p* = 0.1) (Figure 2C).

In 82 IIIB‐IV patients who relapsed after treatment, there was no significant difference in PFS between baseline ctDNA *EGFR*‐negative and *EGFR*‐positive patients regardless of treatment regimen (11.0 months vs. 9.0 months, *p* = 0.1) (Figure 2D). Fifty‐eight of 82 patients with tissue *EGFR*‐positive received first‐ or second‐generation EGFR‐TKIs treatment and 24 of 82 patients with tissue *EGFR*‐negative received chemotherapy. The mPFS of ctDNA *EGFR*‐negative and *EGFR*‐positive patients in the targeted therapy group was 14.0 months vs. 11.0 months (*p* = 0.1), and that was 6.5 months vs. 6.5 months (*p* = 0.5) in the chemotherapy group (Figure 2E,F).

## 4 DISCUSSION

EGFR‐TKI has been widely used in the treatment of advanced NSCLC, but the precondition of EGFR‐TKI application is whether *EGFR* mutation has been determined. Genetic testing based on tumor tissue is considered the gold standard method, However, it was reported that only 18% of advanced NSCLC patients have sufficient tumor specimens for full tissue genotyping of all eight guideline‐recommended genomic biomarkers, thus new alternative tissue samples are needed for genetic testing in NSCLC in clinical practice.^30^, ^31^ Based on real‐world retrospective data, this study analyzed the concordance rate of *EGFR* mutation detection in paired tissue samples and blood samples. Based on the highly sensitive SuperARMS‐PCR method to detect plasma ctDNA *EGFR* mutation, the concordance rate with tissue detection was as high as 79.5%.^32^ In this study, we further analyzed the correlation between baseline plasma ctDNA *EGFR* mutation status and prognosis in advanced patients. Stratified analysis of the treatment status of patients, compared to the intention‐to‐treat population, treatment naïve patients with baseline plasma *EGFR*‐negative would have better PFS which was slightly higher than previously reported.^33^ A trend for longer PFS in baseline ctDNA *EGFR*‐negative patients was also found in relapsed patients. So precise stratification of patients' treatment status can better benefit clinical patients. And since these data were obtained from real clinical practice, it has greater significance for clinical diagnosis and treatment guidance.

Two plasma ctDNA *EGFR* detection methods were included in this study, ARMS‐PCR and SuperARMS‐PCR, because the SuperARMS‐PCR method was available after the year 2018.^34^ SuperARMS‐PCR is more sensitive for mutation detection in cfDNA due to its modified primers and DNA polymerase.^35^, ^36^ In the present study, based on the results of ARMS‐PCR detection, the sensitivity of plasma ctDNA *EGFR* mutation detection was only 59.7%, which was lower than those detection results of SuperARMS‐PCR (68.4%) as well as the published data.^37^ The superiority of SuperARMS‐PCR in detecting ctDNA *EGFR* mutation was more prominent in plasma samples of I‐IIIA patients (the detection sensitivity of SuperARMS‐PCR was 33.3%, while the detection sensitivity of ARMS‐PCR was 0%). This further illustrated the advantages of SuperARMS in the detection of plasma samples with low abundance.^38^ Advanced patients who have progressed on therapy, especially those with EGFR‐TKIs therapy, often carry the T790M resistance mutation, which was more frequently detected in liquid biopsies than in tissues.^39^, ^40^ In the present study, the sensitivity and specificity of ctDNA mutation detection rate by SuperARMS in EGFR TKIs relapsed patients were 76.2% and 60.0%, which were 67.1% and 100.0% in treatment naïve patients. The Relapsed patients had higher sensitivity but worse specificity for mutation detection in ctDNA compared to newly diagnosed patients, resulting in a lower concordance rate than the latter due to novel mutation sites in relapsed patients. Two relapsed patients with T790M co‐mutation was detected in plasma but not in tissue.^39^, ^40^, ^41^ The specificity for relapsed patients in this study in the SuperARMS‐PCR detection group was 75%, also due to the fact that T790M was only detected in plasma samples in two patients. The detection sensitivity of SuperARMS‐PCR ctDNA *EGFR* in relapsed patients was lower than that of ARMS‐PCR, but there was no statistical difference, which may be caused by the low number of samples between the two groups. To further analyze the factors affecting the detection rate of *EGFR* mutation in SuperARMS‐PCR ctDNA, the patient's pathological subtype was an influencing factor. In this study, the consistent rate of ctDNA *EGFR* detected by SuperARMS‐PCR in patients with non‐adenocarcinoma was higher than that in patients with adenocarcinoma. This phenomenon has also been mentioned in other studies.^20^, ^42^

In patients with advanced lung cancer, what is the prognostic value of baseline blood ctDNA mutation status in addition to guiding targeted drugs? Patients from the FLAURA study with undetectable plasma *EGFR* mutations at baseline had better EGFR‐TKI efficacy than patients with detectable plasma *EGFR* mutations at baseline.^13^ The first is the EURTAC trial, the study mentioned that compared with patients with L858R mutation both in tissue and plasma, median OS was longer in those the mutation was detected in a tissue but not in plasma (median OS, 27.7 vs 13.7 months).^43^ In the AURA3 study, PFS was prolonged in patients with tissue T790M‐positive and plasma T790M‐negative results compared with patients with plasma T790M‐positive results both in osimertinib (median, 12.5 vs 8.3 months) and platinum‐pemetrexed groups (median, 5.6 vs 4.2 months).^44^ Gu et al. enrolled 117 acquired TKI resistance advanced NSCLC patients. It was found that patients with T790M detected in plasma had poor OS compared to that T790M un‐detected (median OS, 26.9 months vs. NA).^45^ Elizabeth Fabre et al. also found that baseline ctDNA positivity was associated with reduced OS (median, 13.6 vs.21.5 months) and poor PFS (median, 4.9 vs 10.4 months), those detected by NGS.^15^ These clinical studies confirmed that both treatments naïve and relapsed patients had a worse prognosis if their baseline ctDNA *EGFR* mutation was detected.

In our study, the median duration of follow‐up was 15.0 months (IQR 0.33–140.0). Since the patients in this study were treated with chemotherapy or targeted monotherapy, and the first‐generation or second‐generation EGFR TKIs was used in targeted therapy, the median follow‐up time of 15 months could track more than 50% of patients' relapse.^46^ For treatment‐naïve patients, regardless of whether they received targeted therapy (mPFS 7.0 months vs.14.0 months, *p* = 0.004) or chemotherapy (mPFS 4.0 months vs. 7.0 months, *p* = 0.14), patients with baseline ctDNA *EGFR* mutation had worse PFS. No difference (*p* = 0.1) in the PFS was observed in the chemotherapy group because of the few number of ctDNA *EGFR*‐positive patients (N = 4). Among the relapsed patients who received targeted therapy, patients with plasma ctDNA *EGFR*‐positive before treatment also had worse PFS compared with patients with plasma ctDNA *EGFR*‐negative (11.0 months vs. 14.0 months, p = 0.1). The patients with detectable plasma ctDNA mutation at baseline maybe have a high tumor load, caused by spatial heterogeneity of the tumor, leading to a poor clinical outcome.^47^

There were several limitations in this study. First, due to the limited sample size, a more detailed analysis of the detection rate of *EGFR* mutation sites has no statistical value, so a stratified analysis of *EGFR* classic mutation sites and rare mutation sites was not performed. Second, when analyzing the prognostic value of baseline ctDNA mutation status, PFS was used as the study endpoint rather than OS due to the limited follow‐up time. The survival status of these patients will be followed to obtain OS data. Finally, more patients receiving chemotherapy and immunotherapy will be included in follow‐up studies to observe the correlation between baseline ctDNA *EGFR* mutation status and patient prognosis.

## 5 CONCLUSIONS

This study confirmed that plasma samples can be used as a surrogate for molecular testing when tissue samples were not available in real‐world studies, especially in patients with advanced NSCLC. Compared with the ARMS‐PCR detection method, the SuperARMS‐PCR detection method had higher sensitivity for the detection of ctDNA *EGFR* mutation in plasma. And in patients with stage IIIB‐IV, baseline plasma ctDNA *EGFR* mutation positivity not only guides targeted therapy but also predict a worse prognosis.

## AUTHOR CONTRIBUTIONS

**Chaolian Long:** Conceptualization (equal); data curation (equal); formal analysis (equal); investigation (equal); methodology (equal); project administration (equal); supervision (equal); visualization (equal); writing – original draft (equal); writing – review and editing (equal). **Kun Li:** Conceptualization (equal); data curation (equal); formal analysis (equal); investigation (equal); methodology (equal); project administration (equal); supervision (equal); visualization (equal); writing – original draft (equal); writing – review and editing (equal). **Zichen Liu:** Formal analysis (equal); investigation (equal); visualization (equal). **Nana Zhang:** Formal analysis (equal); investigation (equal); visualization (equal). **Xuya Xing:** Formal analysis (equal); investigation (equal); visualization (equal). **Liming Xu:** Data curation (equal); formal analysis (equal); methodology (equal). **Fei Gai:** Data curation (equal); formal analysis (equal); methodology (equal). **Nanying Che:** Conceptualization (lead); data curation (lead); formal analysis (lead); funding acquisition (equal); investigation (lead); methodology (lead); project administration (lead); resources (lead); supervision (lead); validation (lead); visualization (lead); writing – review and editing (lead).

## FUNDING INFORMATION

This work was supported by grants from the National Natural Science Foundation of China (Grant Numbers: 82072381), Beijing Municipal Science and Technology Project (Grant Numbers: Z181100001918027, Z191100006619079).

## CONFLICT OF INTEREST

Liming Xu and Fei Gai are full‐time employees of Amoy Diagnostics Co, Ltd.

## ETHICAL APPROVAL

The study was approved by the Ethical and Institutional Review Boards for Human Investigation of the Beijing Chest Hospital (number: 2020‐keyan‐linshen14).

## Supporting information


Figure S1.

Table S1.

## Data Availability

Some or all data generated or used during the study are available from the corresponding author by request.
