# Supplementary figures and images for: Real‐world analysis of the prognostic value of EGFR mutation detection in plasma ctDNA from patients with advanced non‐small cell lung cancer
Source: Cancer Med. 2023 Jan 9;12(7):7982–91. doi: 10.1002/cam4.5582 (PMC10134383; doi:10.1002/cam4.5582)

A

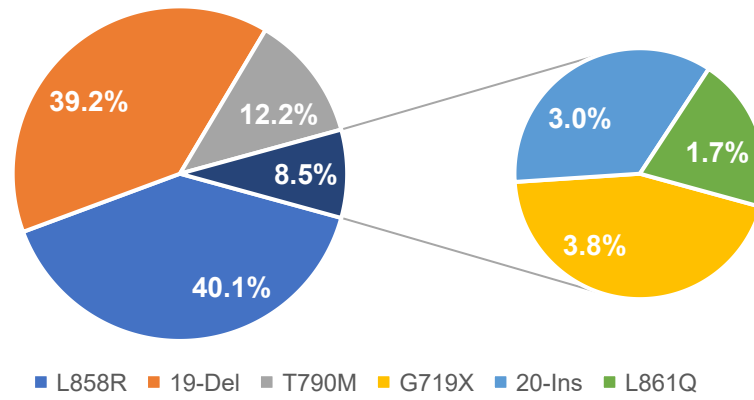

B

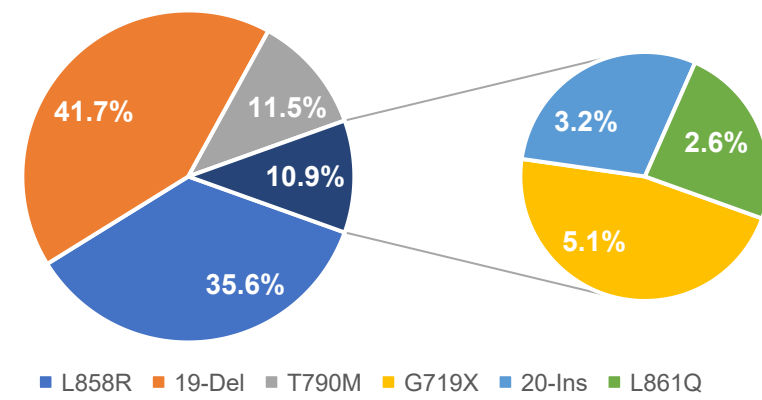

Supplement: Supplementary file 1 — Figure S1. [file CAM4-12-7982-s001.pdf]
