# Supplementary material for: Real‐world analysis of the prognostic value of EGFR mutation detection in plasma ctDNA from patients with advanced non‐small cell lung cancer
Source: Cancer Med. 2023 Jan 9;12(7):7982–91. doi: 10.1002/cam4.5582 (PMC10134383; doi:10.1002/cam4.5582)
Supplement: Supplementary file 2 — Table S1. [file CAM4-12-7982-s002.docx]

**Table S1. The plasma ctDNA detection performance from samples detected by SuperARMS grouped by pathological type**

|  |  | **Sensitivity** | **Specificity** | **PPV** | **NPV** | **concordance** |
| --- | --- | --- | --- | --- | --- | --- |
|  |  |  |  |  |  |  |
| **Pathological type** | Adenocarcinoma | 67.2%  (58.1%,75.2%) | 96.8%  (87.8%,99.4) | 97.7%  (91.1%,99.6%) | 59.4%  (49.2%,68.9%) | 77.0%  (70.5%,82.5%) |
|  |  |  |  |  |  |  |
|  | Non-adenocarcinoma | 87.5%  (46.7%,99.3%) | 100.0%  (80.0%,100.0%) | 100.0%  (56.1%,100.0%) | 95.2%  (74.1%,99.8%) | 96.4%  (82.3%,99.4%) |

**Table S2. The plasma ctDNA detection performance from samples detected by SuperARMS grouped by treatment history**

|  |  | **Sensitivity** | **specificity** | **PPV** | **NPV** | **Concordance** |
| --- | --- | --- | --- | --- | --- | --- |
|  |  |  |  |  |  |  |
| **Treatment naïve patients** | | 67.1%  (55.5%,77.0%) | 100.0%  (93.9%,100.0%) | 100.0%  (91.6%,100.0%) | 74.0%  (64.1%,82.0%) | 83.00%  (72.3%,88.1%) |
|  |  |  |  |  |  |  |
| **Relapsed patients** | ALL | 70.4%  (56.2%,81.6%) | 75.0%  (35.6%,95.5%) | 95.0%  (81.8%,99.1%) | 27.3%  (11.6%,50.4%) | 71.0%  (58.7%,80.8%) |
|  | Relapsed from adjuvant chemotherapy | 50.0%  (22.3%,77.7%) | 100.0%  (31.0%,100.0%) | 100.0%  (51.7%,100.0%) | 33.3%  (9.0%,69.1%) | 60.0%  (35.8%,80.2%) |
|  |  |  |  |  |  |  |
|  | Relapsed from EGFR TKIs | 76.2%  (60.2%,87.4%) | 60.0%  (17.0%,92.7%) | 94.1%  (78.9%,99.0%) | 23.1%  (6.2%,54.0%) | 74.5%  (60.5%,84.8%) |
